# Reply to Scarlata, G.G.M.; Abenavoli, L. Comment on “Rotaru et al. Lean MASLD and IBD: Exploring the Intersection of Metabolic Dysfunction and the Gut–Liver Axis. *Life* 2025, *15*, 288”

**DOI:** 10.3390/life15081211

**Published:** 2025-07-30

**Authors:** Adrian Rotaru, Remus Stafie, Ermina Stratina, Sebastian Zenovia, Robert Nastasa, Horia Minea, Laura Huiban, Tudor Cuciureanu, Cristina Muzica, Stefan Chiriac, Irina Girleanu, Ana-Maria Singeap, Catalin Sfarti, Carol Stanciu, Anca Trifan

**Affiliations:** 1 Department of Gastroenterology, Grigore T. Popa University of Medicine and Pharmacy, 700115 Iasi, Romania; adrianrotaru94@yahoo.com (A.R.); stratina.ermina@yahoo.com (E.S.); sebastianzenovia20@gmail.com (S.Z.); robertnastasa948@gmail.com (R.N.); horia.minea@yahoo.com (H.M.); huiban.laura@yahoo.com (L.H.); drcuciureanutudor@gmail.com (T.C.); lungu.christina@yahoo.com (C.M.); gilda_iri25@yahoo.com (I.G.); anamaria.singeap@yahoo.com (A.-M.S.); cvsfarti@gmail.com (C.S.); stanciucarol@yahoo.com (C.S.); ancatrifan@yahoo.com (A.T.); 2 Institute of Gastroenterology and Hepatology, “St. Spiridon” University Hospital, 700111 Iasi, Romania

We are grateful to Dr. Scarlata and Prof. Abenavoli for their insightful and collegial remarks on our recent publication [1,2]. Their engagement highlights the importance of continued academic dialogue around the emerging clinical relevance of metabolic dysfunction-associated steatotic liver disease (MASLD) in patients with inflammatory bowel disease (IBD). We fully share their view on the relevance of reclassifying non-alcoholic fatty liver disease under the MASLD terminology, and we appreciate their emphasis on the gut–liver axis as a central pathophysiological mechanism linking these two conditions [3].

We fully endorse their view that the recent terminological transition from NAFLD to MASLD represents more than a nomenclatural change—it reflects a deeper understanding of the cardiometabolic underpinnings of steatotic liver disease, including in patients traditionally considered metabolically healthy by anthropometric measures. In this regard, their pilot study significantly advances the field by demonstrating an 18% prevalence of MASLD among Italian IBD patients—substantially higher than NAFLD (6%)—and by correlating MASLD with increased disease activity and higher rates of surgical intervention. These findings are congruent with our own clinical observations and mechanistic hypotheses [4]. We also wish to highlight the results of a recent large cohort study by Zhang et al. which confirmed a significantly elevated all-cause mortality risk in IBD patients with MASLD, particularly in the lean MASLD subgroup (HR = 3.14). This finding further emphasizes the urgent need for early hepatic screening in IBD populations regardless of BMI or traditional metabolic risk factors [5].

Also, over the past years, our group has devoted substantial energy and academic focus to elucidating the burden of steatotic liver disease in patients with IBD. In our prospective study published in Journal of Clinical Medicine [6], we were the first to systematically screen a Romanian IBD cohort using vibration-controlled transient elastography with controlled attenuation parameter, documenting a prevalence of NAFLD of 46.3%—a figure significantly higher than that reported in many previous European studies. Beyond merely reporting prevalence, we demonstrated that liver steatosis in IBD is not confined to patients with obesity or overt metabolic syndrome. In our cohort, nearly half of the patients with steatosis had a normal BMI, underscoring the relevance of lean MASLD, a phenotype now gaining increasing recognition. Moreover, we identified independent associations between hepatic steatosis and age, BMI, CRP, disease duration, fasting glucose, triglycerides, and type 2 diabetes, reflecting both inflammatory and metabolic drivers. A quarter of our patients also exhibited significant fibrosis (≥F2), reinforcing the silent but progressive nature of liver injury in IBD.

Taken together, the converging evidence from our group and colleagues such as Abenavoli and Scarlata strongly argues for an updated clinical framework that integrates hepatologic screening into routine IBD care. This approach should transcend BMI thresholds and instead focus on metabolic and inflammatory profiles. It is increasingly clear that lean individuals are not exempt from MASLD or its complications. We appreciate the authors’ call for multidisciplinary management and agree that future prospective studies should explore how emerging IBD therapies—including biologics—may modulate hepatic outcomes, particularly in lean MASLD phenotypes.

## Data Availability

The data presented in this study are available upon request from the corresponding author. The data are not publicly available because they are the property of the Institute of Gastroenterology and Hepatology, Iasi, Romania.

## Abbreviations

The following abbreviations are used in this manuscript:

IBD	Inflammatory bowel disease
NAFLD	Nonalcoholic Fatty Liver Disease
MASLD	Metabolic Dysfunction-associated Steatotic Liver Disease
BMI	Body Mass Index
CRP	C-Reactive Protein
